# Supplementary material for: Backbone Effects on the Thermoelectric Properties of Ultra-Small Bandgap Conjugated Polymers
Source: Polymers (Basel). 2021 Jul 28;13(15):2486. doi: 10.3390/polym13152486 (PMC8347066; doi:10.3390/polym13152486)
Supplement: Supplementary file 1 [file polymers-13-02486-s001.zip › polymers-1283147-SI.pdf]

Supporting Information

Backbone Effects on the Thermoelectric Properties of Ultra-small Bandgap Conjugated Polymers

De-Xun Xie[1,2,†], Jing Xiao[1,2,†], Quanwei Li[1,2], Tongchao Liu[3], Jinjia Xu[4], and Guang Shao[1,2,*]

1) School of Chemistry, Sun Yat-sen University, Guangzhou, 510275, China;

2) Shenzhen Research Institute, Sun Yat-sen University, Shenzhen, 518057, China.

3) Shenzhen Key Laboratory of Polymer Science and Technology, College of Materials Science and Engineering, Shenzhen University, Shenzhen 518060, China.

4) Weldon School of Biomedical Engineering, Purdue University, West Lafayette, IN, 47906, USA.

* Correspondence: shaog@mail.sysu.edu.cn

† De-Xun Xie and Jing Xiao contributed equally to this work.

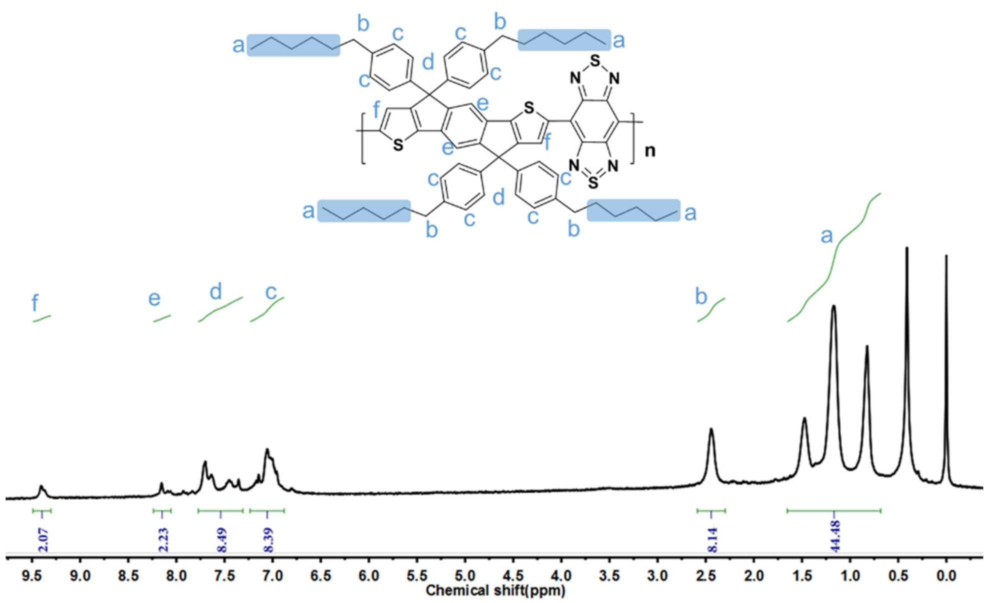

(a)

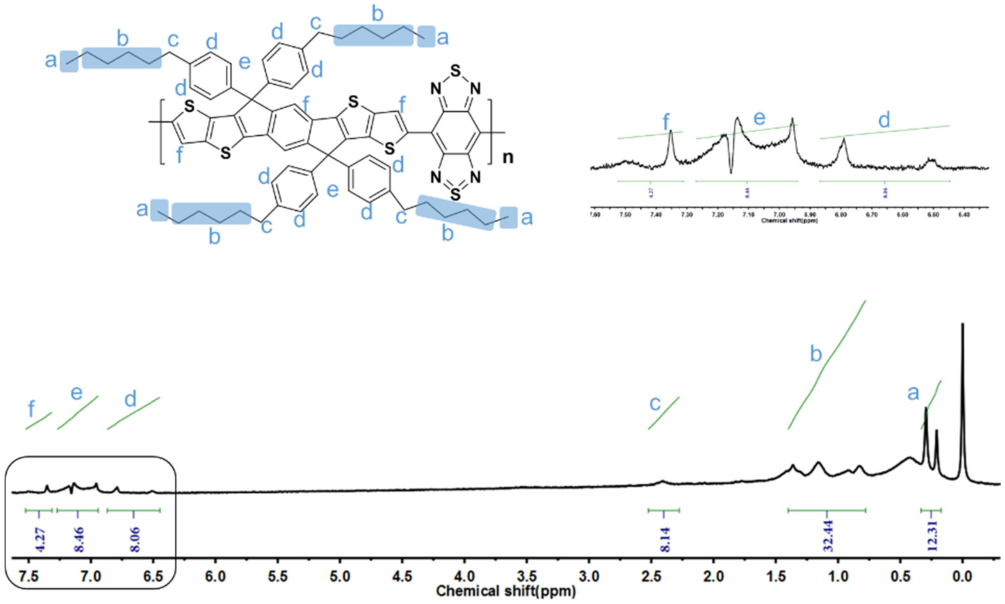

(b)

Figure S1. ^{1}H NMR spectra of (a) PIDT-BBT and (b) PIDTT-BBT.

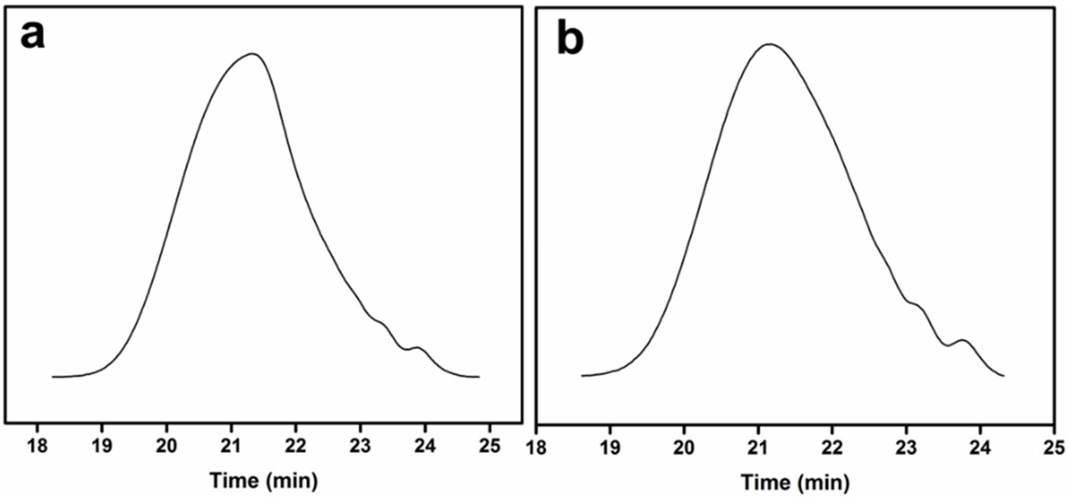

Figure S2. GPC curve of (a) PIDT-BBT and (b) PIDTT-BBT.

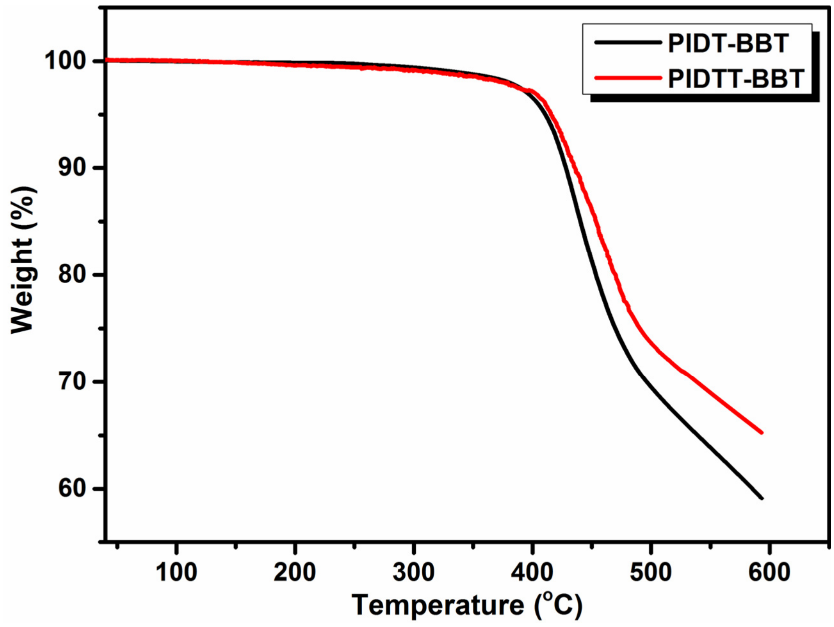

Figure S3. TGA curve of PIDT-BBT and PIDTT-BBT.

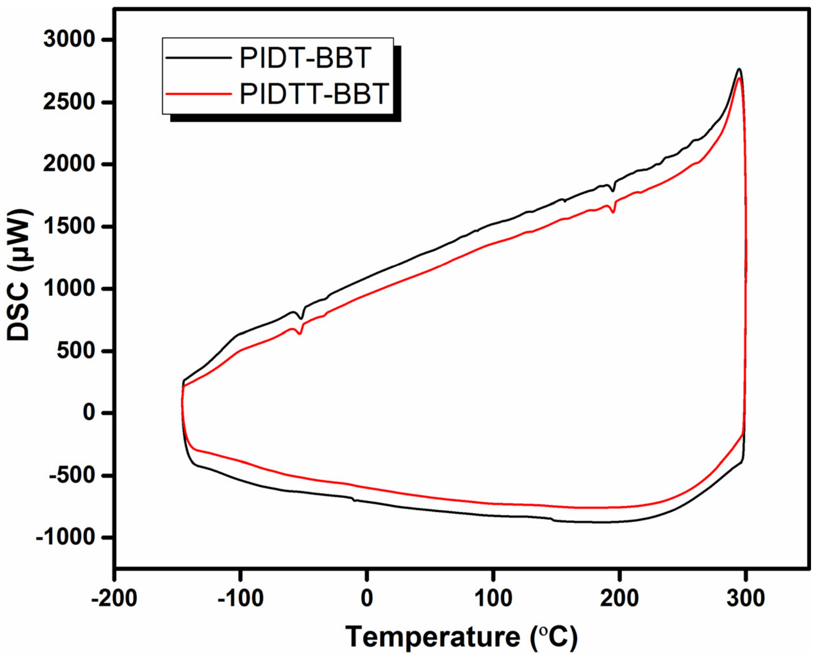

Figure S4. DSC spectra of PIDT-BBT and PIDTT-BBT.

Table S1. Molecular weights and thermal properties of PIDT-BBT and PIDTT-BBT.

Polymer	M_n (KDa)	M_w (KDa)	PDI	n	T_g (°C)	T_d (°C)
PIDT-BBT	16.85	44.32	2.63	15	146	410
PIDTT-BBT	16.76	39.91	2.38	14	138	415

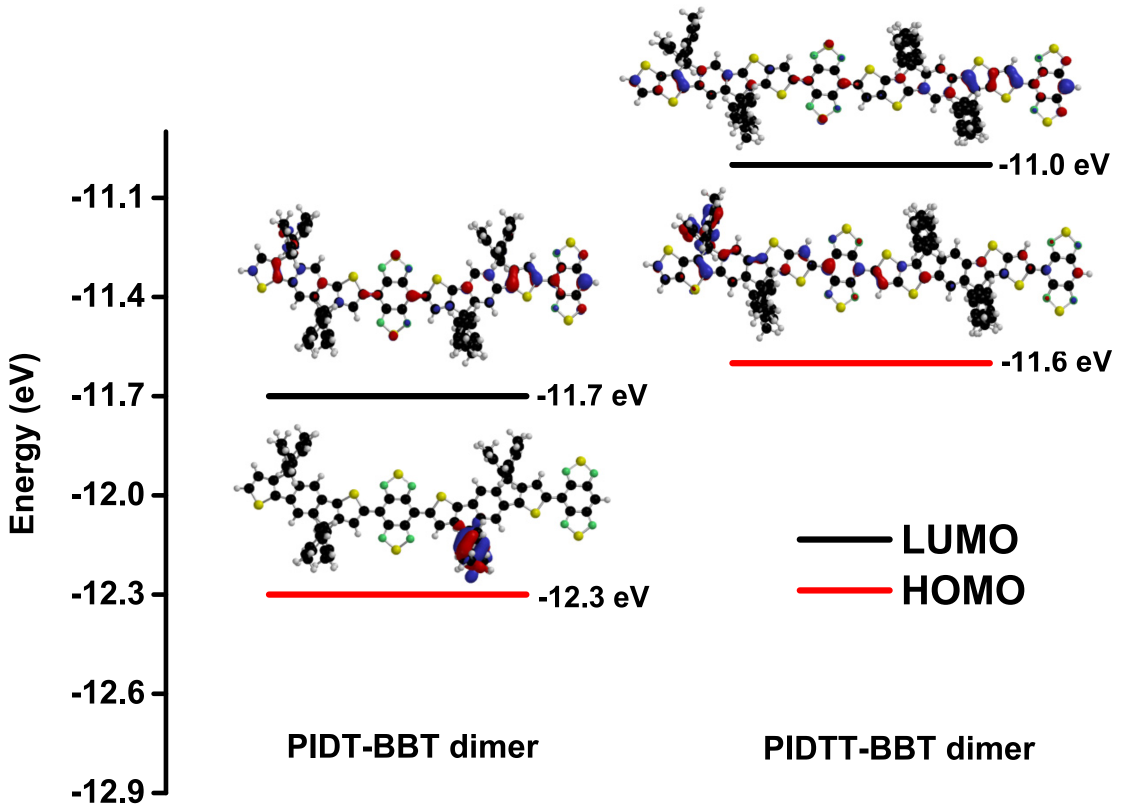

Figure S5. Calculated HOMO/LUMO molecular orbitals (B3LYP/6-31G* levels) for the dimers of PIDT-BBT and PIDTT-BBT.

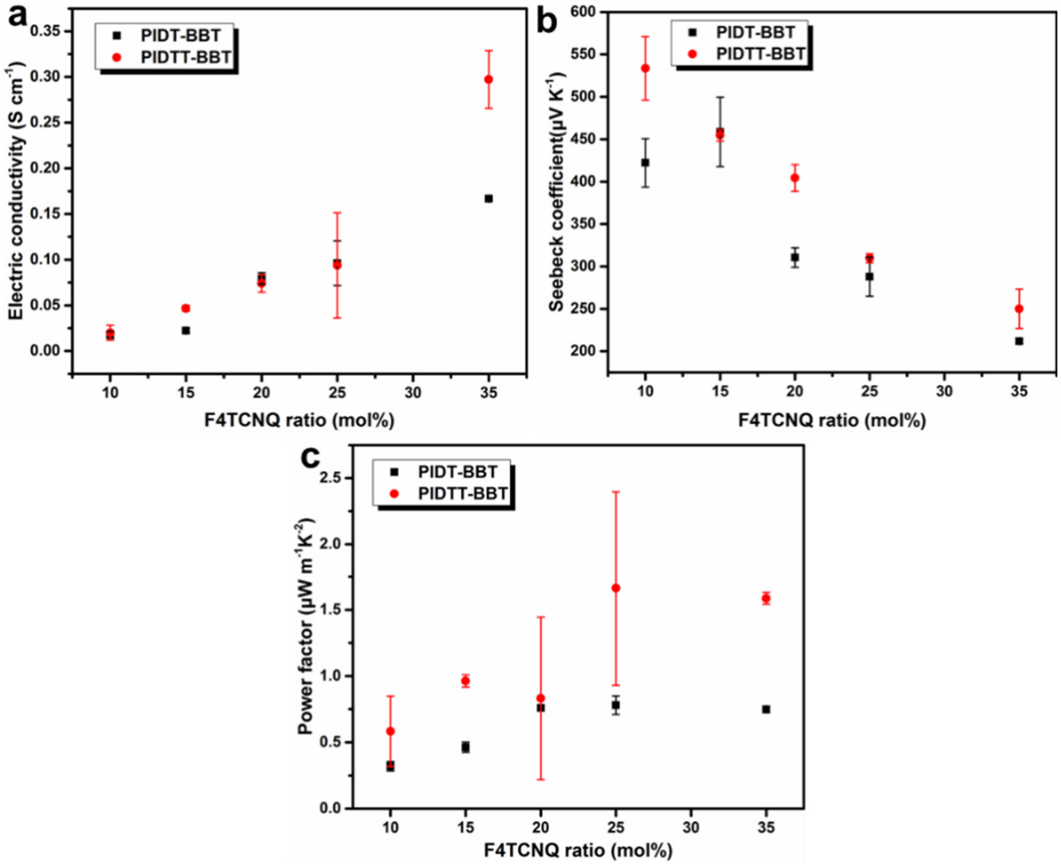

Figure S6. Thermoelectric performance of the polymers doped with different F4TCNQ molar ratios: (a) electrical conductivity $\boldsymbol{\sigma}$, (b) Seebeck coefficient S, and (c) power factor PF.

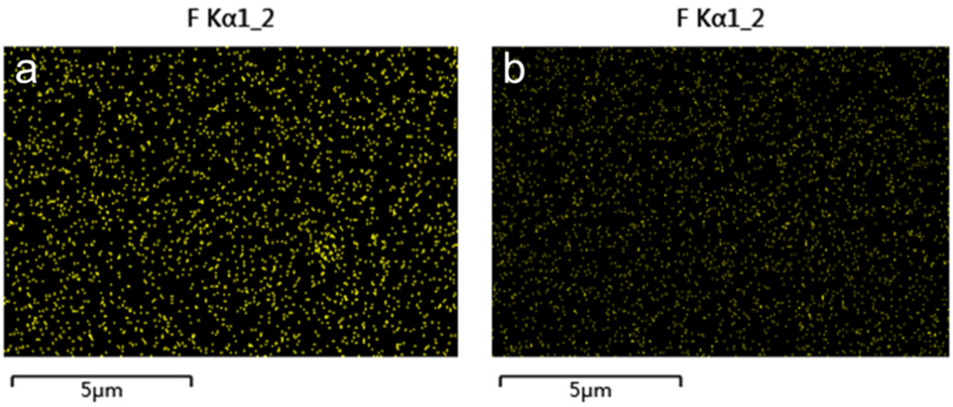

Figure S7. EDS of (a) PIDT-BBT and (b) PIDTT-BBT films doped with F4TCNQ (25 mol%).

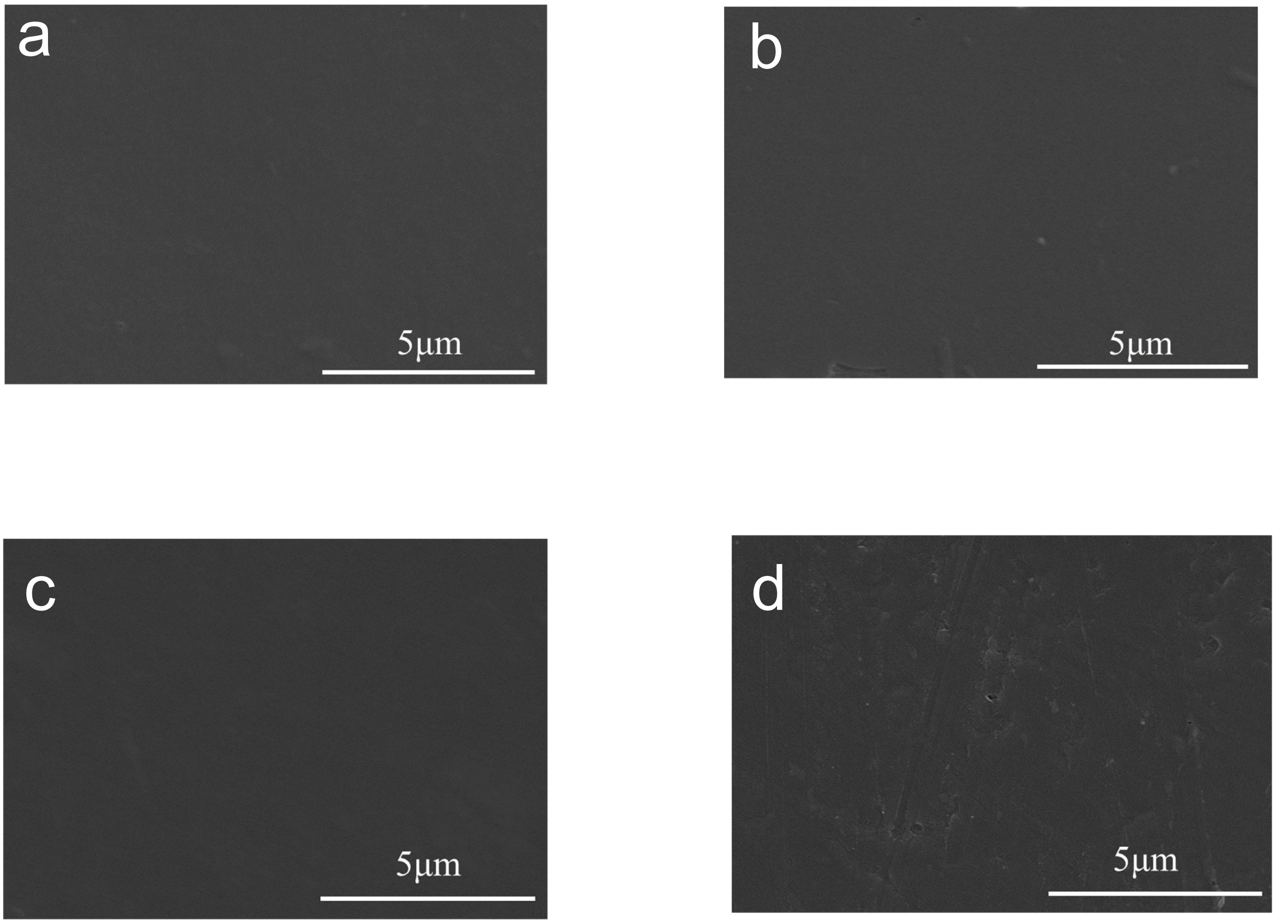

Figure S8. SEM images of PIDT-BBT (a) pristine films and (b) films doped with F4TCNQ, PIDTT-BBT (c) pristine films and (d) films doped with F4TCNQ.

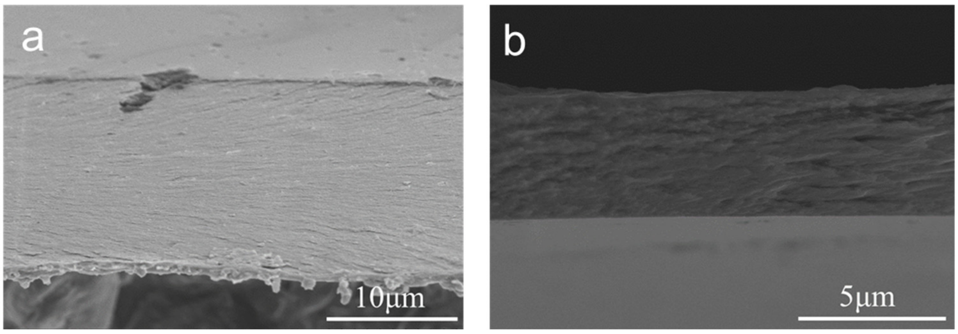

Figure S9. Cross-sectional SEM images of pristine (a) PIDT-BBT and (b) PIDTT-BBT films.

Details of the FET Fabrications and Testing: Solution-processed films of polymer are fabricated by dip coating method. SAM-modified substrate is immersed in the polymer solution (5 mg/mL) in a mixed solvent of dichloromethane and acetone (1:1 by volume), and subsequently pulling up at a constant speed of 10 μm/s as controlled by a LongerPump TJ-3A syringe pump controller. Vacuum-deposited films of the polymer solution are deposited on SAM-modified AlO_x using an vacuum coating system with a Turbomolecular pump (OPV450, SKY Technology Development Co., Ltd., CAS) at a pressure of 2.0×10^{-6} torr or lower, with a deposition rate of ca. 1 Å/s to a thickness of 40 nm as measured by a quartz crystal sensor. OTFTs of BTBT-C12 are completed by depositing a layer of gold through a shadow mask onto the dip-coated or vacuum-deposited films to form top-contact source and drain electrodes. The field effect mobilities of these OTFTs in the saturation regime are measured under ambient conditions and extracted from transfer *I-V* curves using the equation $I_{DS} = (\mu W C_i/2L)(V_{GS}–V_T)^2$, where I_{DS} is the drain current; μ is field effect mobility; C_i is the capacitance per unit area for the SAM-modified AlO_x dielectrics; W is the channel width; L is the channel length; V_{GS} and V_T are the gate and threshold voltages, respectively. The current-voltage measurement of thin-film transistors is conducted using a probe station (PS-100, Lakeshore) and a Keithley 4200A Semiconductor Characterization System in air under ambient conditions.
